# Reply to Maase et al. Comment on “Jones et al. Application of a Novel Algorithm for Expanding Newborn Screening for Inherited Metabolic Disorders across Europe. *Int. J. Neonatal Screen.* 2022, *8*, 20”

**DOI:** 10.3390/ijns9010008

**Published:** 2023-02-16

**Authors:** Simon A. Jones, David Cheillan, Anupam Chakrapani, Heather J. Church, Simon Heales, Teresa H. Y. Wu, Georgina Morton, Patricia Roberts, Erica F. Sluys, Alberto Burlina

**Affiliations:** 1 Willink Biochemical Genetics Unit, Manchester Centre for Genomic Medicine, Manchester University NHS Foundation Trust, St Mary’s Hospital, Oxford Road, Manchester M13 9WL, UK; 2 Service Biochimie et Biologie Moléculaire, Groupement Hospitalier Est, Hospices Civils de Lyon, 69002 Lyon, France; 3 Department of Metabolic Medicine, Great Ormond Street Hospital NHS Foundation Trust, London WC1N 3JH, UK; 4 Neurometabolic Unit, University College London Hospitals NHS Foundation Trust and Enzymes Laboratory, Great Ormond Street Hospital NHS Foundation Trust, London WC1N 3JH, UK; 5 ArchAngel MLD Trust, Registered Charity No. 1157825, London W2 1DT, UK; 6 Helvet Health, Ruelle de la Muraz 4, 1260 Nyon, Switzerland; 7 Division of Inherited Metabolic Diseases, Reference Centre Expanded Newborn Screening, University Hospital Padova, 35128 Padova, Italy

The commentary provided by Maase et al. [1] demonstrates how the novel algorithm [2] could be utilised by countries to evaluate disorders through expanded newborn screening programmes. We read with interest the detail provided about the Dutch evaluation method and appreciate the similarities described in their point-based scoring approach. We thank the authors for their comments.
